# Soil Matrix Modulates Polystyrene Microplastic Toxicity to *Eisenia fetida*: From Acute Lethality to Oxidative Genotoxicity

**DOI:** 10.3390/cimb48080780

**Published:** 2026-07-30

**Authors:** Xiaoling Xu, Chuanxiang Li, Haochen Sun, Jian Wang, Jinbo Liu

**Affiliations:** School of Petroleum Engineering and Environmental Engineering, Yan’an University, Yan’an 716000, China; xuxiaoling0309@163.com (X.X.); 15634129251@163.com (C.L.); 18829781399@163.com (H.S.); wangjian@yau.edu.cn (J.W.)

**Keywords:** polystyrene, microplastics, earthworm, artificial soil, natural soil, toxicity

## Abstract

Microplastic (MP) contamination in soils has raised widespread concern, yet the extent to which soil matrices regulate MP toxicity remains insufficiently characterized. In this study, the acute and subchronic toxicity of polystyrene (PS) to the earthworm (*Eisenia fetida*) was analyzed in artificial soil and three types of natural soils (lou soil, black soil and latosol soil). The 28 d LC_50_ (50% lethal concentration) of PS in artificial soil was 130.86 g kg^−1^, whereas it decreased to 39.71 and 39.23 g kg^−1^ in lou and latosol soil, respectively, and increased to 175.13 g kg^−1^ in black soil, indicating that soil physicochemical properties may modulate MP toxic potency. Across all soil types, PS exposure triggered reactive oxygen species (ROS) accumulation in earthworms, which in turn activated antioxidant and detoxification enzymes (superoxide dismutase, catalase, and glutathione S-transferase) and ultimately led to lipid peroxidation and DNA damage. Assessment via the integrated biomarker response index suggested divergent PS toxicity across soils, indicating inconsistencies between artificial soil and natural soils. Notably, this study was conducted under controlled laboratory conditions using a single polymer type, particle size, and test organism, and therefore the observed soil-dependent toxicity patterns may not be directly extrapolated to other MP types, sizes, or soil biota. Overall, our study suggested that the toxicity data derived from artificial soil cannot fully represent the real effects in natural soils, providing valuable insights for elucidating the realistic toxicity of MPs.

## 1. Introduction

Microplastics (MPs) represent a ubiquitous environmental pollutant class [1,2,3,4,5,6]. Soils are widely identified as their primary terrestrial sink, with contamination stemming from plastic mulch use, sewage sludge amendment, and atmospheric deposition [7]. Unlike aquatic environments, soil matrices present a complex heterogeneous system where MPs interact intimately with soil biota [8,9,10]. While the ubiquity of MPs is well-documented [7,8], the specific ecological risks they pose to terrestrial organisms remain a critical area of investigation. MPs can be transported across different environmental compartments, accumulated along food chains, and ingested by organisms at various trophic levels [11,12]. Furthermore, because of their high surface reactivity, MPs can adsorb metals and organic pollutants, thereby amplifying their potential health hazards [13,14]. Therefore, systematic investigations into the biological effects of MPs on terrestrial organisms are of great theoretical and practical significance.

Earthworms, as ecosystem engineers, play a pivotal role in maintaining soil structure and function [15,16]. Owing to their direct contact with soil particles and sensitivity to xenobiotics, earthworms (e.g., *Eisenia fetida*) are standard bioindicators for assessing soil health [17,18]. Exposure to MPs can trigger a cascade of physiological responses in earthworms, including oxidative stress, immune responses, and energy metabolism disorders [19,20]. However, the severity of these toxicological outcomes is heavily modulated by the soil matrix itself. Soil properties such as organic matter content, particle size, and pH can alter the bioavailability of MPs [21], yet these interactions are often oversimplified in standard toxicity assays. While the general principle that soil properties modulate contaminant toxicity is well established in ecotoxicology, a critical knowledge gap remains regarding the validity of the standard artificial soil protocol for assessing the toxicity of MPs. Current regulatory guidelines for soil toxicity testing predominantly rely on artificial soil to ensure reproducibility [20]. However, a significant knowledge gap exists regarding the extrapolation of these laboratory results to complex natural environments. Artificial soil lacks the intricate organo-mineral associations and aged organic matter (e.g., ultralaminae) found in natural soils, which can sequester contaminants and reduce their bioaccessibility [21]. Consequently, toxicity data derived from artificial substrates may misrepresent the actual risks in natural ecosystems [22]. It remains unclear how soil-specific properties mediate the toxicity of MPs across different natural soil types compared with standard artificial soil.

To address these gaps, we conducted a comparative toxicity assessment of polystyrene (PS, a polymer commonly encountered in the environment [23]) in artificial soil and three representative natural soils (lou soil, black soil, and latosol soil). To quantify acute lethal toxicity, we calculated 28-day LC_50_ values based on earthworm mortality rates across PS exposure gradients. Reactive oxygen species (ROS), superoxide dismutase (SOD), catalase (CAT), glutathione S-transferase (GST), malondialdehyde (MDA) and 8-hydroxydeoxyguanosine (8-OHdG) were measured to evaluate the subchronic toxic effects. The toxicity difference in PS between artificial and natural soils was assessed using the integrated biomarker response (IBR) method. We hypothesized that earthworm response in artificial and natural soil differ substantially, and toxicity data generated from artificial soil assays cannot reliably mirror PS-induced physiological harm in natural earthworm populations. This study fills an important knowledge gap regarding the soil-dependent toxicity of MPs, and suggests that the toxic effects observed in artificial soils cannot represent those in natural soils, thereby providing valuable scientific support for more accurate and environmentally relevant risk evaluations of MP contamination.

## 2. Materials and Methods

### 2.1. Reagents and PS

The fluorescent probe 2′,7′-dichlorohydro fluorescein diacetate (DCFH-DA, purity > 97%) was purchased from Aladdin Biochemical Co., Ltd., Shanghai, China. The PS microplastics utilized in the current experiment matched the material specifications described in our prior work [24]. The PS had an average size of 24.65 ± 5.20 μm and an organic carbon content of 0.92 g g^−1^.

### 2.2. Preparation of Samples

Four soil substrates with divergent physicochemical properties were selected for toxicity testing. Three kinds of natural soils, including lou soil, black soil and latosol soil (Eum-Orthic Anthrosol, USDA), were obtained from the Shaanxi, Heilongjiang, and Hainan Provinces of China. Standard OECD artificial soil (70% quartz sand, 20% kaolin clay, 10% peat moss) was prepared following the official OECD test guideline. The physicochemical properties of the soils were determined using standard methods, and the specific methods and results are presented in Text S1 and Table S1 (Supplementary Material), respectively.

Healthy adult earthworms (300–600 mg) were purchased from the Hebei Earthworm Breeding Centre. Prior to exposure, they were pre-cultured in the four prepared soils for 24 h in an incubator (RGD-1000B-LED, Ningbo, China).

### 2.3. PS Toxicological Experiment Design

**Figure 1:**
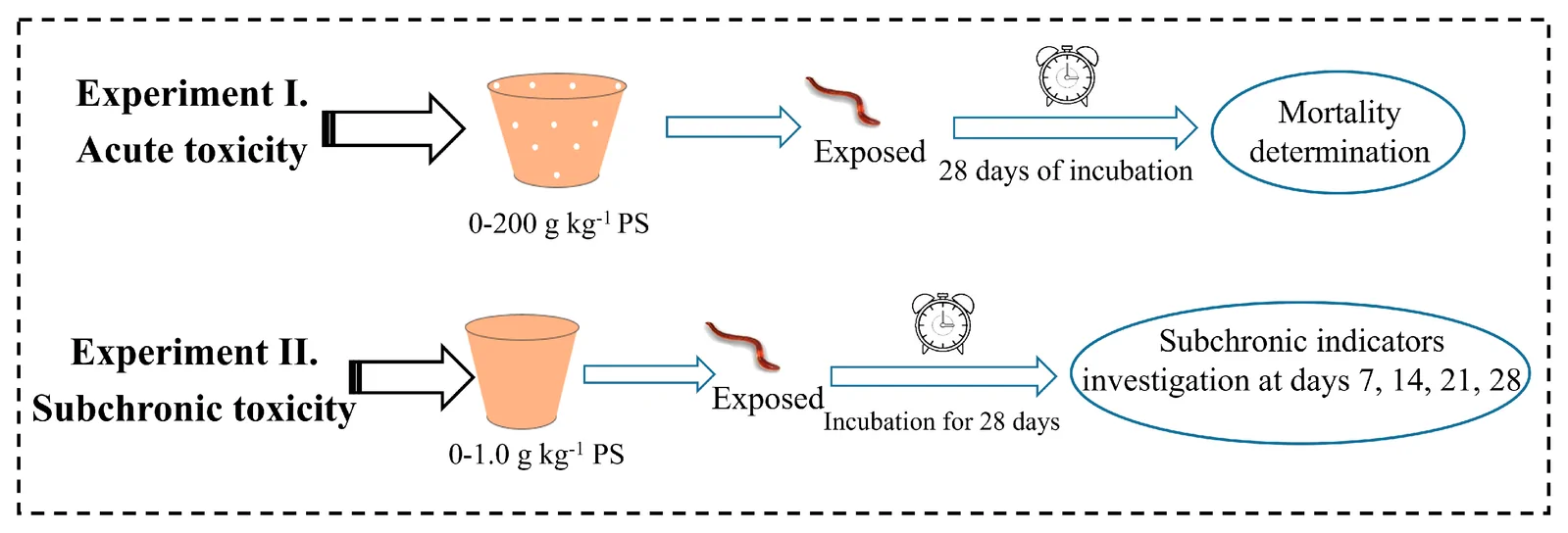
Experimental design.

The experimental flowchart is shown in Figure 1, and the details are provided below.

#### 2.3.1. Acute Toxicity

The acute toxicity of PS was assessed using pot experiments. Specifically, PS was added to soil at concentrations of 0, 10, 25, 50, 100, and 200 g kg^−1^. After a 2 h equilibration period, 30 adult earthworms were randomly allocated to each experimental pot to eliminate handling-related bias. Each replicate (9 replicates) was housed in an independent container with separate soil matrices, ensuring true biological replication and independence. The placement of these containers in the incubator was randomized and rotated regularly to avoid positional effects. Throughout the incubation period, soil moisture was maintained at 60% of the maximum water-holding capacity. Mortality was recorded after 28 days. The above high-concentration gradient was primarily designed to calculate 28 d LC_50_ values and define the lethal toxicity threshold of PS microplastics across distinct soil matrices, which provides fundamental benchmark data for comparing the maximum tolerable exposure doses of earthworms in different soil environments. However, the concentration range applied in this acute trial far exceeds the MP levels commonly detected in actual farmland soils. To complement this limitation and strengthen the environmental relevance, a supplementary acute test with environmentally relevant PS concentrations was additionally performed. In this assay, PS concentrations were set at 0, 5, 10, 20, 25, and 50 g kg^−1^, and earthworm mortality was recorded after 7 days of exposure.

#### 2.3.2. Subchronic Toxicity

Subchronic exposure experiments followed the same core setup as the acute toxicity trials. However, PS concentrations were settled at 0, 0.1, 0.25, 0.5, and 1.0 g kg^−1^ based on the 0.25% (*w*/*w*) environmental content [25]. Subsequently, earthworms were added, and individuals were randomly selected for toxicity measurements at days 7, 14, 21, and 28. Due to the limited tissue biomass yielded by single earthworm specimens, tissues harvested from three individuals within one biological replicate were combined to generate sufficient material for biochemical quantification. Each pooled sample was treated as a single independent biological replicate.

### 2.4. Detection of Biomarkers of Subchronic Toxicity

#### 2.4.1. ROS Content

The ROS concentration was detected via DCFH-DA. In detail, earthworms were washed, dried, weighed, homogenized in phosphate-buffered saline (0.1 M, pH = 7.4), and the homogenate was subsequently centrifuged at 20,000× *g* for 20 min at 4 °C. Next, the pellet was collected and resuspended to prepare the mitochondria suspension, and then 0.1 mL DCFH-DA was added and reacted for 0.5 h. Finally, the reaction was quenched by adding 0.2 mL of 1 M HCl. Fluorescence was measured using a Varioskan LUX multifunctional microplate reader (Thermo Fisher Scientific, Waltham, MA, USA) at excitation and emission wavelengths of 488 nm and 525 nm, respectively.

#### 2.4.2. Activities of Antioxidant and Detoxification Enzymes and MDA Content

In this experiment, earthworms were also homogenized in phosphate-buffered saline and then centrifuged for 20 min. The resulting supernatants were collected to quantify SOD, CAT, GST, and MDA activities in strict accordance with the manufacturer’s protocols (Suzhou Keming Biotechnology Co., Ltd., Suzhou, China).

#### 2.4.3. Evaluation of Coelomocytes DNA Damage

The 8-OHdG was detected using a corresponding earthworm 8-OHdG ELISA kit (Shanghai Hengyuan Company, Shanghai, China). The earthworms were suspended through phosphate-buffered saline at 4 °C, then centrifuged for 20 min. Subsequently, following the instruction of the corresponding assay kit, the optical density of the supernatants was measured at 450 nm using a microplate reader, and the results were used to calculate the concentration of 8-OHdG.

### 2.5. Bioavailability and Uptake of PS

To quantify the bioavailability of PS in different soils, soil pore water was extracted after 7 days of exposure following a standardized stepwise extraction protocol. Each soil sample was transferred into a pressure soil pore water sampler, and pore water was collected under consistent suction pressure. The collected pore water samples were transferred to centrifuge tubes and centrifuged at 10,000 rpm for 20 min at room temperature to precipitate soil colloids and large mineral particles. The resulting supernatant was vacuum-filtered through a 0.45 μm nitrocellulose membrane filter to retain all labile-free PS microplastics present in the pore water. After filtration, each filter membrane was carefully peeled from the filter holder, placed in a sealed glass Petri dish, and stored in a dark, dust-free cabinet at 4 °C before microscopic counting to avoid particle loss and contamination. The entire filter surface was systematically scanned under an optical microscope (Model BX53, Olympus Corporation, Hachioji-shi, Tokyo, Japan) at 400× magnification. Random non-overlapping visual fields (*n* = 15 fields per filter) were selected for particle enumeration; only intact PS fragments with clear spherical morphology were counted, while irregular mineral impurities were excluded based on morphological distinction. The total PS particle number across all fields was extrapolated to calculate the full-filter particle abundance, which was then converted to pore water PS concentration, expressed as particles per liter of water (items/L).

Meanwhile, the particle size distribution of recovered PS from bulk soil matrices was analyzed via density flotation and SEM observation using standardized procedures. Specifically, approximately 10 g homogenized soil sample was transferred into a 500 mL glass beaker containing 100 mL saturated ZnCl_2_ solution (density = 1.5 g cm^−3^). The mixture was magnetically stirred for 10 min at 300 rpm to fully suspend PS particles from soil aggregates. After that, the suspension was transferred to centrifuge tubes and centrifuged for 10 min at 8000× *g* to separate low-density PS into the supernatant. The supernatant was vacuum-filtered through a 0.25 μm cellulose membrane to trap the separated PS particles. The filters loaded with PS were placed in covered glass Petri dishes and air-dried at room temperature for 48 h to remove residual salt solution before SEM preparation. The dried filter membranes were cut into small uniform slices, fixed on conductive copper stubs, and sputter-coated with a 10 nm gold layer to enhance electrical conductivity. The samples were observed under a scanning electron microscope (Nova Nano SEM-450, FEI, Hillsboro, OR, USA) at an accelerating voltage of 10 kV. For each soil treatment, a minimum of 200 intact discrete PS particles were randomly captured across multiple imaging positions to avoid sampling bias. The diameter of each captured PS particle was measured using Nano Measurer software(Nano Measurer 2.0). Mean particle size and size distribution frequency were calculated for each soil group. For SEM particle size quantification, a minimum of 200 intact discrete PS particles were randomly captured across multiple imaging positions per soil replicate. Only intact spherical PS fragments with clear boundary outlines were selected for diameter analysis; heavily aggregated particle clusters and mineral impurities were excluded to eliminate measurement bias. One-way ANOVA combined with LSD post hoc testing was used to compare mean particle diameters across the four soil substrates.

To determine the uptake of PS in earthworm tissues, a complete standardized protocol including depuration, digestion and PS recovery was adopted. Specifically, after 7 days of PS exposure, earthworms were collected, rinsed with ultrapure water, and placed individually on moistened dust-free filter paper in sterile plastic boxes for 24 h depuration at 25 °C. Fresh filter paper was replaced every 12 h to prevent re-ingestion of egested soil and PS particles, ensuring complete evacuation of gut contents. The depurated earthworms were rinsed three times with ultrapure water, surface moisture was gently blotted with lint-free filter paper, and individual fresh weights were recorded before digestion. Whole earthworm tissues were transferred into glass digestion tubes, mixed with 10 mL 30% H_2_O_2_ solution, and incubated in a 60 °C water bath for 72 h until all soft organic tissues were fully dissolved with no visible tissue residues. The cooled digestate was mixed with saturated ZnCl_2_ solution, fully vortexed, and left to stand for 2 h to float PS particles out of the digested organic residues. The upper flotation supernatant was vacuum-filtered through a 0.45 μm nitrocellulose membrane, and the retained PS particles were enumerated under the Olympus BX53 microscope (Olympus Corporation, Tokyo, Japan) following the same field-selection and morphological screening rules as pore water samples. Bioaccumulation levels were calculated and expressed as the number of PS particles per gram of earthworm fresh weight (items/g).

These supplementary tests conducted during revision (7 d short-term acute toxicity assay, pore water PS extraction and bioavailability quantification, earthworm tissue PS digestion and accumulation measurement, SEM particle morphology observation, post-exposure particle size distribution analysis) strictly adopted the same experimental frameworks as the original acute/subchronic toxicity trials. Identical PS microplastic stock suspension, four pre-treated soil substrates, adult *Eisenia fetida* individuals, incubation temperature (25 ± 1 °C), light–dark cycle, soil water-holding capacity (60% field maximum water capacity), randomization rules, biological replicate settings, earthworm depuration procedures, homogenization buffers, digestion reagents, detection kits and instrument calibration standards were applied throughout all experiments. No differences in cultivation, sample pretreatment or testing protocols existed between the original and supplementary measurements, ensuring comparability of all datasets.

### 2.6. Statistical Analysis

The data of pore water PS bioavailability, tissue PS accumulation, SEM particle characterization, post-exposure particle size distribution, the supplementary 7-day acute toxicity test, and the three-way ANOVA statistical results were acquired and analyzed exclusively during the manuscript revision. None of these datasets existed or were measured at the time of original submission. These measurements, sample characterizations and multivariate statistical modeling were specially designed, conducted and processed entirely during the revision phase. All data were analyzed using SPSS 22.0 and visualized using Origin 2025. A three-way analysis of variance (three-way ANOVA) was applied to quantify the independent and interactive effects of three fixed factors including soil type, PS exposure concentration, and exposure duration on measured subchronic toxic biomarkers (ROS, SOD, CAT, GST, MDA, 8-OHdG). Prior to the three-way ANOVA implementation, two diagnostic tests were conducted on all biomarker datasets to validate parametric statistical assumptions. The Shapiro–Wilk normality test confirmed that all response variables (ROS, SOD, CAT, GST, MDA, and 8-OHdG) met the criteria for normal distribution. In addition, Levene’s test indicated that the assumption of variance homogeneity was not violated across any of the soil × concentration × time treatment combinations. Thus, the data satisfied the prerequisites for parametric statistical analysis. These results confirm that parametric three-way ANOVA is statistically appropriate for the dataset. Type III sum of squares was adopted to account for minor unbalanced replicate counts, and partial eta-squared (η^2^_p_) was calculated as the standardized effect size to quantify the relative explanatory power of each main and interactive factor. The residual degrees of freedom for all models equal 640. Data for indicators following treatment with PS at a dose of 0.1 g kg^−1^ was collected and used to determine the IBR index, and this index was applied to measure the toxicity among the various soils following the method of Sanchez et al. [26]. The detailed calculation process is provided in Text S2.

## 3. Results

### 3.1. Acute Toxicity of PS

The acute toxicity of PS to *Eisenia fetida* was evaluated using the 28 d LC_50_ index (Table 1). As expected, mortality was positively correlated with the PS, and the 28 d LC_50_ in artificial soil was 130.86 g kg^−1^ (95% CIs, 88.15 to 254.27 g kg^−1^). However, the response of earthworms to PS varied substantially among the other soil types. In lou soil, black soil and latosol soil, the 28 d LC_50_ values were 39.71, 175.13 and 39.23 g kg^−1^, respectively. The 28 d LC_50_ values highlighted that the acute toxicity of PS varied considerably across soil types, meaning PS acute toxicity in lou and latosol soils was approximately four-fold greater than in artificial soil. Consistent with these findings, the 7 d LC_50_ values obtained from the low-dose short-term trial exhibited a similar trend (Table S2). This shorter-term exposure trial adopted a narrower, environmentally aligned concentration gradient (0–50 g kg^−1^) to mirror real farmland MP pollution. This low, field-relevant concentration gradient independently validated the soil-mediated toxicity trends identified in the high-dose 28-day lethal assay, indicating that matrix-induced differences in PS toxicity remain detectable under environmentally realistic contamination levels.

### 3.2. ROS Accumulation

**Figure 2:**
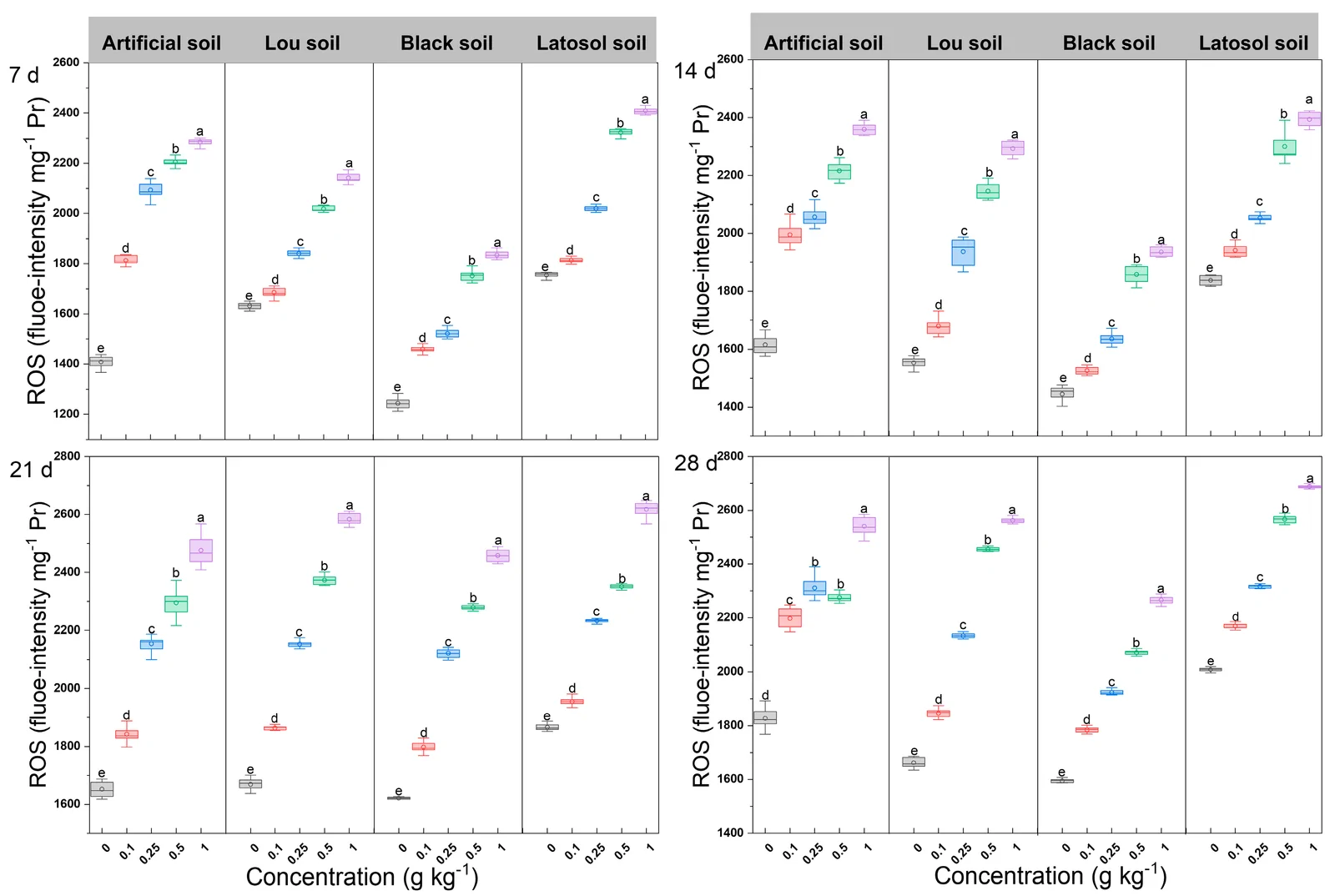
The concentration of reactive oxygen species (ROS) in earthworms following polystyrene (PS) exposure at various doses in different soils. Error bars represent standard error of the mean (*n* = 9). Significant differences among treatments were analyzed via one-way ANOVA followed by LSD test. Distinct lowercase letters above bars denote significant differences at *p* < 0.05.

ROS are key indicators of early oxidative stress in organisms under pollutant exposure. The production of ROS in earthworms exposed to PS for 7–28 days was measured using DCFH-DA fluorescence (Figure 2). Across all soil types, PS exposure induced a significant increase in ROS levels compared with unamended controls (*p* < 0.05), indicating the onset of oxidative stress. After 7 days of exposure in artificial soil, ROS levels were significantly higher than in the untreated control soil. On day 7, ROS fluorescence intensity increased linearly when dose of PS enhanced up to 0.25 g kg^−1^, but no further significant increase was observed when the exposure dose was raised from 0.5 to 1.0 g kg^−1^. A similar result was observed for lou soil, black soil, and latosol soil amended with PS. Moreover, ROS content in four soils increased obviously from 7 to 28 days compared with the control groups following exposure to PS. In artificial soil, lou soil, latosol soil, earthworms exposed to 1.0 g kg^−1^ PS exhibited peak ROS fluorescence intensity of 2540, 2562 and 2689 fluoe-intensity mg^−1^ Pr, respectively, on day 28. In black soil, that max value appeared on day 21 (2458 fluoe-intensity mg^−1^ Pr), then decreased to 2266 fluoe-intensity mg^−1^ Pr at day 28.

### 3.3. Responses of Antioxidant and Detoxification Enzymes

#### 3.3.1. SOD and CAT Activities

**Figure 3:**
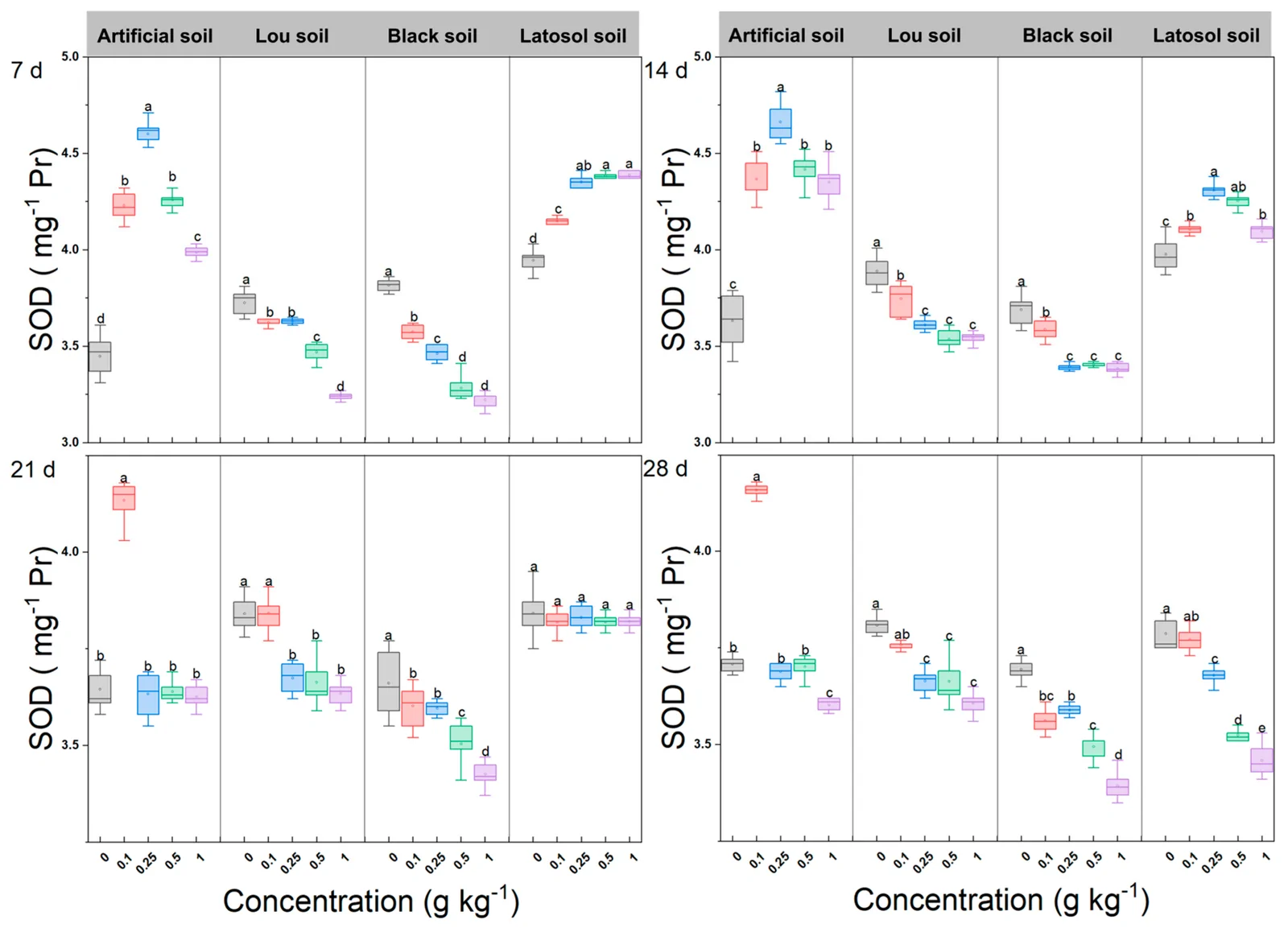
The superoxide dismutase (SOD) activity in earthworms following polystyrene (PS) exposure at various doses in different soils. Error bars represent standard error of the mean (*n* = 9). Significant differences among treatments were analyzed via one-way ANOVA followed by LSD test. Distinct lowercase letters above bars denote significant differences at *p* < 0.05.

SOD and CAT are the first line of the cellular antioxidant defense system. As presented in Figure 3, SOD activity was regulated by both PS concentration and soil type throughout the 28-day exposure period. In artificial soil, exposure to 0.1 g kg^−1^ PS resulted in higher SOD activity compared with that in natural soils throughout the incubation period. Nevertheless, at higher PS dose, SOD activity was higher than in the control within the first 14 days but subsequently decreased significantly. In lou and black soils, SOD activity was significantly lower in PS-treated groups than in controls. In latosol soil, SOD activity was higher during the initial 14 days and then returned to levels similar to the control. These divergent SOD responses demonstrate that PS exerts matrix-dependent antioxidant modulation, which can be attributed to soil physicochemical traits altering total ROS generation in earthworm tissues.

The changes in CAT activity displayed roughly similar trends to those of SOD (Figure S1). Specifically, at low PS concentrations (0.1 and 0.25 g kg^−1^), CAT activity in artificial soil increased during the first 14 days and was subsequently suppressed. Higher PS concentrations (0.5 and 1.0 g kg^−1^) repressed CAT activity, which remained lower than that in the control throughout the exposure period. In lou soil, this index was equal to or higher than that in control soil, except in the 1.0 g kg^−1^ treatment at day 21 and day 28. In black soil, CAT activity increased at days 7 and 14 (for all PS doses) and subsequently decreased. In latosol soil, CAT activity was generally higher than the control, with the exception of the 0.1 g kg^−1^ at days 7 and 28. These results also indicated that CAT activity in earthworms is closely related to the soil types. A general trend is that CAT activity in earthworms exposed to low PS content was not obviously affected, whereas at higher PS concentrations, CAT activity was generally lower (except in latosol soil).

#### 3.3.2. GST Activity

**Figure 4:**
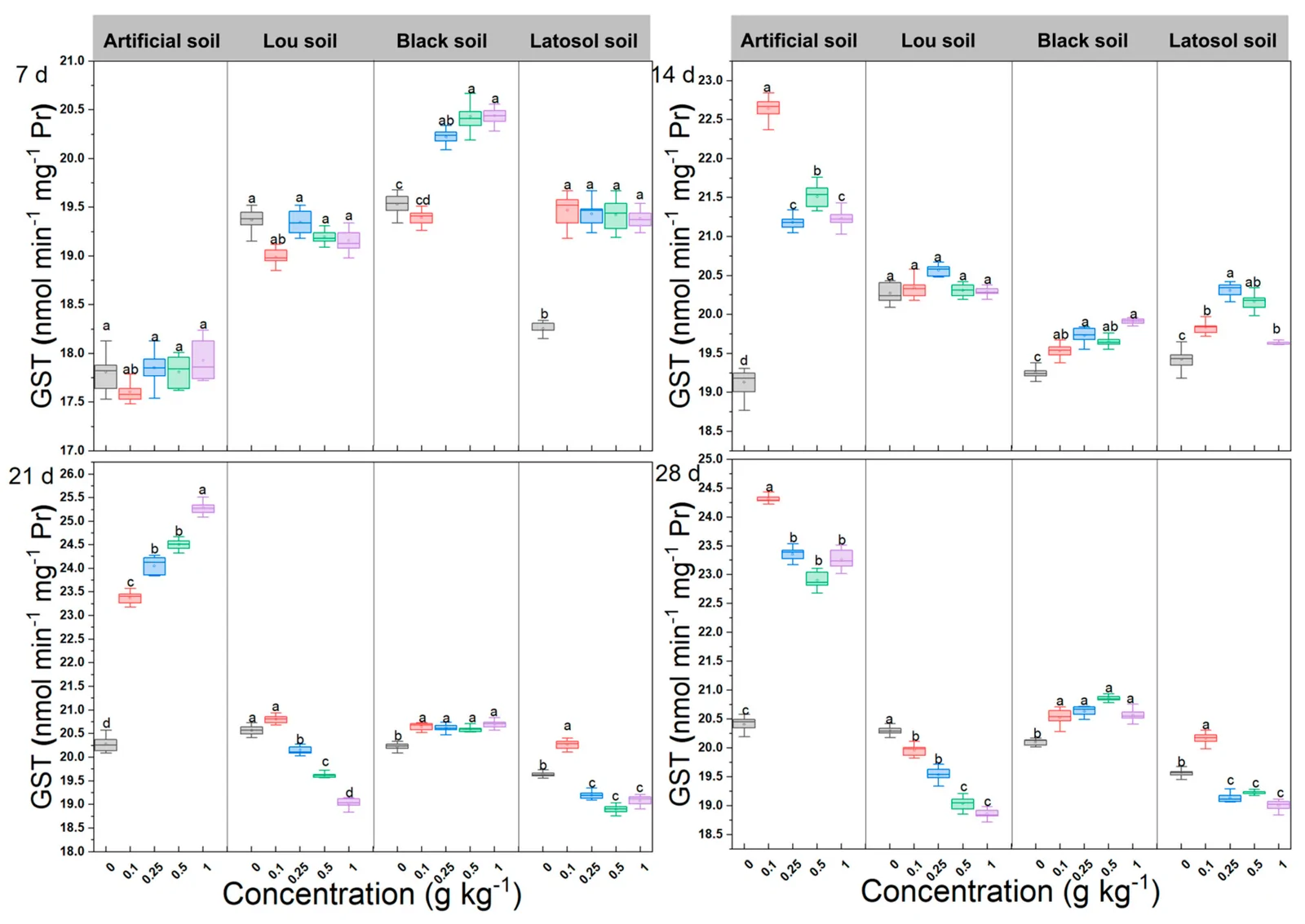
The glutathione S-transferase (GST) activity in earthworms following polystyrene (PS) exposure at various doses in different soils. Error bars represent standard error of the mean (*n* = 9). Significant differences among treatments were analyzed via one-way ANOVA followed by LSD test. Distinct lowercase letters above bars denote significant differences at *p* < 0.05.

GST is a typical enzyme in the detoxification system, and its changes after exposed to PS were investigated and the results are shown in Figure 4. In PS-treated artificial soil, GST activity in earthworms was enhanced after 7 days incubation compared with the control. In lou soil, GST activity was not significantly different from the control soil at day 7 and day 14 and subsequently decreased, except for 0.1 g kg^−1^ at day 21. In black soil, PS exposure induced elevated GST enzymatic activity. In latosol soil, GST activity remained higher after 28 days at a PS dose of 0.1 g kg^−1^, whereas at a higher PS dose, GST activity decreased after 14 days. The varied GST responses further reflected the different stress degrees of earthworms exposed to PS in the four soils.

### 3.4. Lipid Peroxidation and DNA Damage

**Figure 5:**
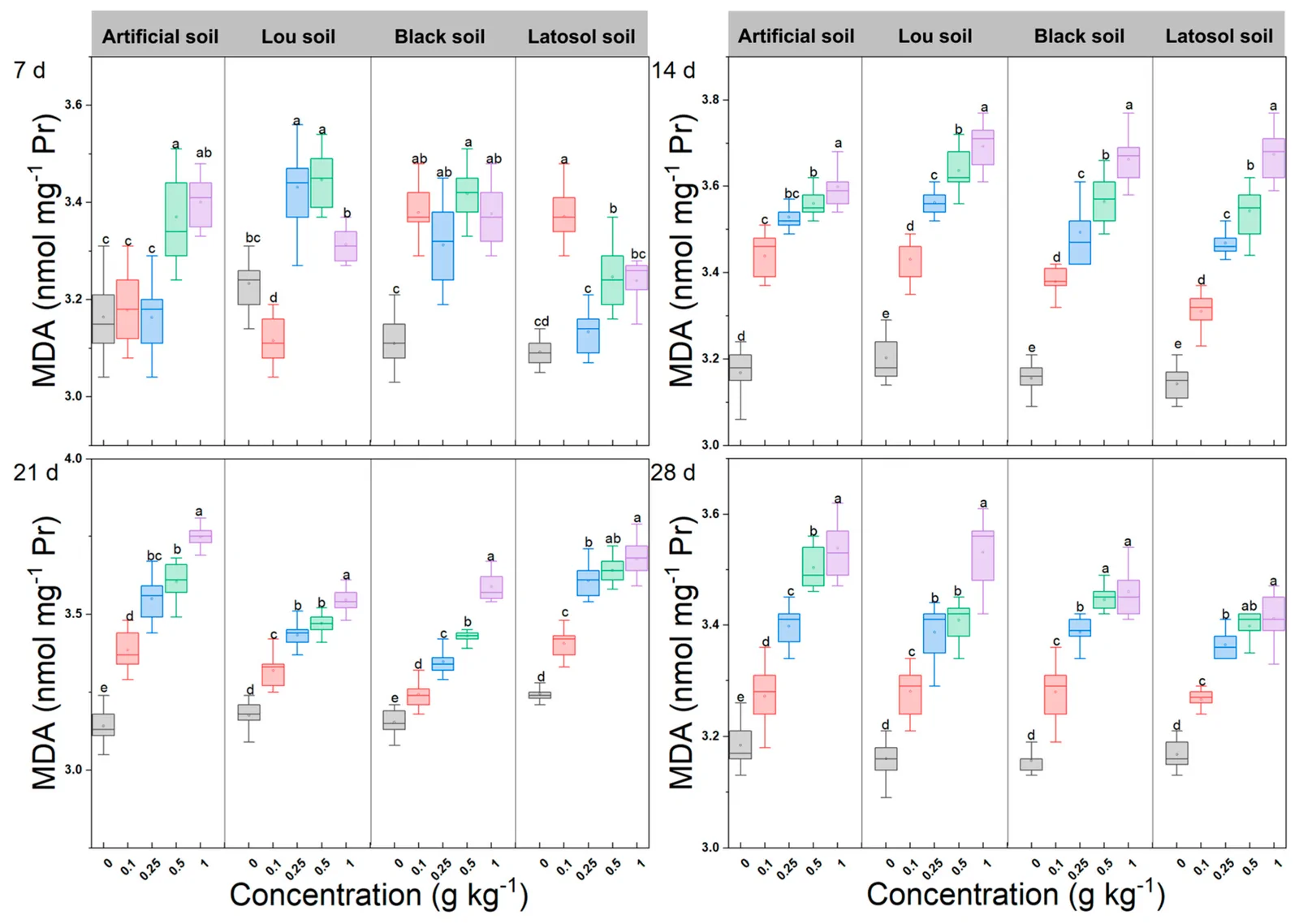
The content of malondialdehyde (MDA) in earthworms following polystyrene (PS) exposure at various doses in different soils. Error bars represent standard error of the mean (*n* = 9). Significant differences among treatments were analyzed via one-way ANOVA followed by LSD test. Distinct lowercase letters above bars denote significant differences at *p* < 0.05.

MDA content can be used to reflect the oxidative damage, and the MDA content in different soils is exhibited in Figure 5. Although MDA content varied among soils during the first 7 days, it increased in all soils under PS exposure from days 14 to 28 relative to the controls. Moreover, MDA level increased with increasing PS concentration in most treatments. Changes in MDA are closely related to the accumulation of ROS in earthworms, leading to lipid peroxidation when the corresponding enzymes are unable to suppress it. Nevertheless, during the incubation, the rate of enzyme production may not keep pace with the increase in ROS content, resulting in more severe lipid peroxidation. In this study, oxidative damage persisted until day 28 after treatment, indicating that the earthworms were affected by oxidation beyond their self-repair capacity. The varying MDA levels among different soils further highlight the significant impact of soil properties on MP-induced oxidative damage, mainly by regulating the bioavailability and toxicity of PS particles. The persistent increase in MDA at the late-exposure stage also reflects a mismatch between ROS production and the antioxidant capacity of *Eisenia fetida*, which is consistent with the inhibited detoxification enzyme activity and indicates sustained cellular damage under MP stress.

**Figure 6:**
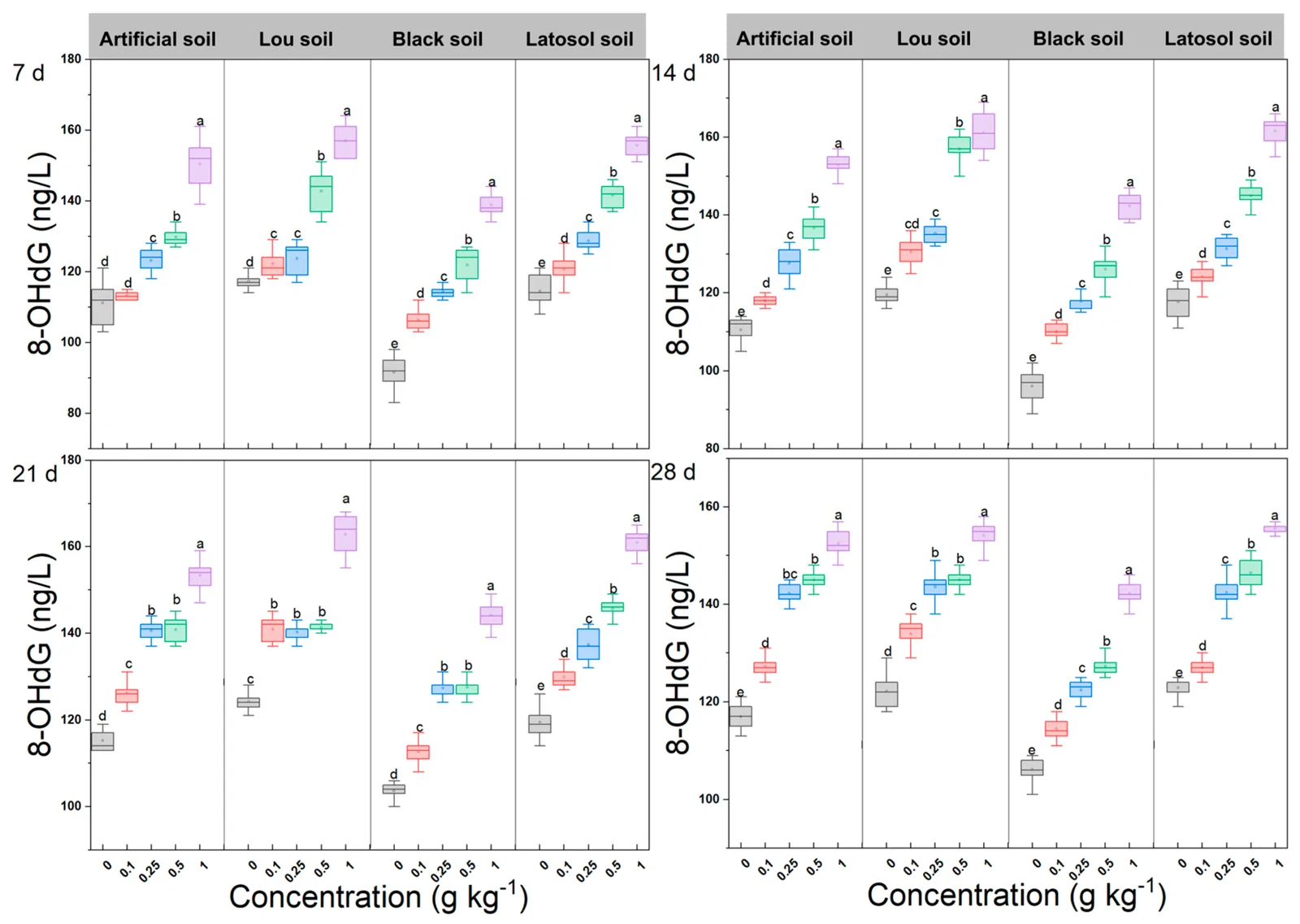
The content of 8-hydroxydeoxyguanosine (8-OHdG) in earthworms following polystyrene (PS) exposure at various doses in different soils. Error bars represent standard error of the mean (*n* = 9). Significant differences among treatments were analyzed via one-way ANOVA followed by LSD test. Distinct lowercase letters above bars denote significant differences at *p* < 0.05.

8-OHdG content was measured to assess DNA damage. As shown in Figure 6, 8-OHdG levels were positively correlated with PS doses in most groups, with values significantly higher than those in the control groups. PS exposure caused oxidative stress (Figure 2) and lead to the generation of ROS, which damaged DNA and generated 8-OHdG. At higher PS exposure levels, higher ROS concentrations were observed in earthworms which in turn enhanced the 8-OHdG level. Notably, the 8-OHdG content induced by PS varied distinctly among the four soil types. Specifically, 8-OHdG level was obviously higher in lou soil and latosol soil than in artificial soil and black soil under the same PS exposure concentration, indicating that PS caused more severe DNA damage to earthworms in these two natural soils. This divergence is primarily attributed to the inherent differences in soil physicochemical properties, which regulate the bioavailability of PS and the ability of earthworms to scavenge ROS and mitigate oxidative damage.

Three-way ANOVA was performed to disentangle the independent and combined contributions of soil type, PS concentration and exposure duration to the oxidative and genotoxic biomarkers (Table S3). For the measured biomarkers (ROS, SOD, CAT, GST, MDA, 8-OHdG), the three main factors all exhibited statistically significant main effects (*p* < 0.05). Among the three variables, soil type consistently produced the largest F-values across all biomarkers, demonstrating that soil matrix served as the dominant driver shaping PS-induced toxic responses, followed by PS concentration and exposure time. All two-way interactive terms (soil type × concentration, soil type × time, concentration × time) and the three-way interaction (soil type × concentration × time) were also significant (*p* < 0.05), indicating that the magnitude of PS toxicity across dose and time gradients was strongly dependent on soil matrix properties.

### 3.5. Bioavailability and Uptake of PS

**Figure 7:**
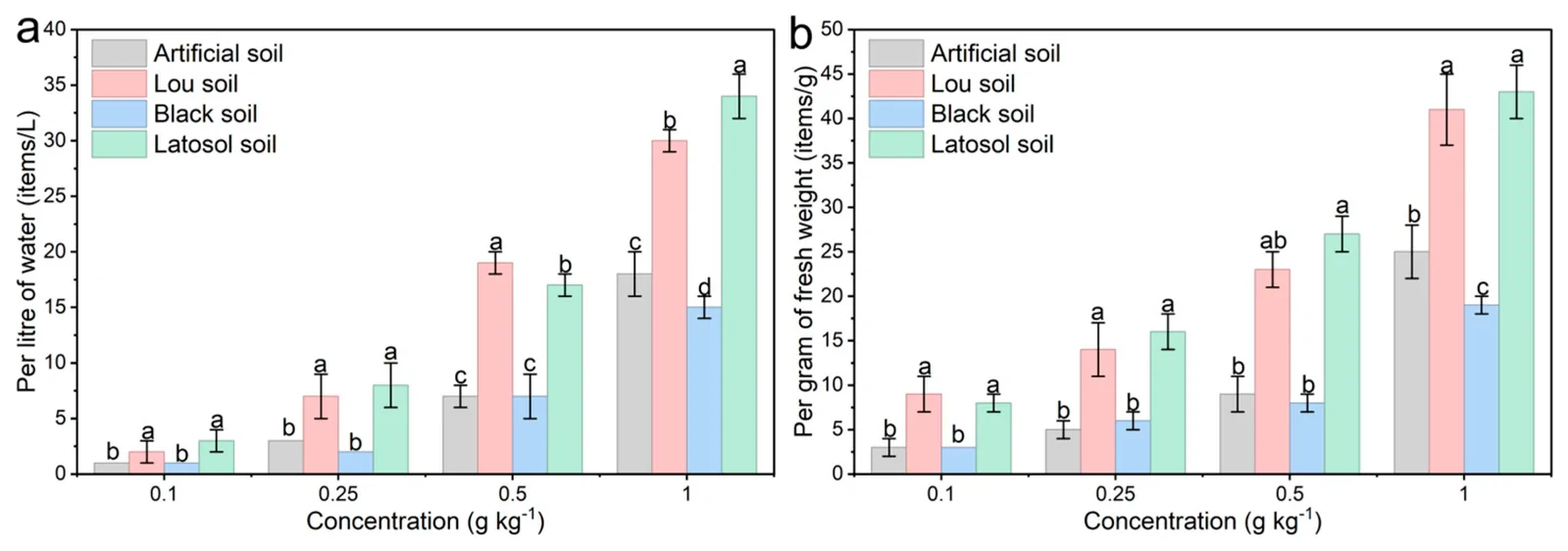
Bioavailability of polystyrene (PS) in soil pore water (**a**) and uptake of PS by earthworms (**b**) across different soil types at varying concentrations. Error bars represent standard error of the mean (*n* = 3). Significant differences among treatments were analyzed via one-way ANOVA followed by LSD test. Distinct lowercase letters above bars denote significant differences at *p* < 0.05.

The concentration of PS in soil pore water, representing bioavailability, exhibited a clear dose-dependent increase across all soil types (Figure 7a and Table S4). The bioavailability of PS in lou soil and latosol soil was significantly higher than that in artificial and black soils (*p* < 0.05), especially for higher PS concentrations (0.5 and 1 g kg^−1^). Specifically, at the highest concentration of 1 g kg^−1^, the PS content in pore water for lou soil (30 items/L) and latosol soil (34 items/L) was markedly higher than that in black soil (15 items/L) and artificial soil (18 items/L). This suggests that the physicochemical properties of lou and latosol soils facilitate greater mobilization or weaker retention of PS particles in the pore water compared with black soil. Similarly, the uptake of PS by earthworms generally mirrored the trends observed in pore water bioavailability (Figure 7b and Table S5). PS uptake increased with increasing soil concentration in all treatments. Earthworms inhabiting lou soil and latosol soil fed significantly higher amounts of PS compared to those in artificial and black soils. At the 1 g kg^−1^ treatment, the uptake in lou soil (41 items/g) and latosol soil (43 items/g) was more than double that observed in black soil (19 items/g). In addition, PS particles recovered from black soil (31.12 ± 1.21 μm) and artificial soil (30.59 ± 2.58 μm) exhibited significantly larger sizes compared with those from lou soil (27.47 ± 3.15 μm) and latosol soil (26.85 ± 2.63 μm) after 7-day exposure (Figure S2).

### 3.6. IBR and Correlation Analysis

**Figure 8:**
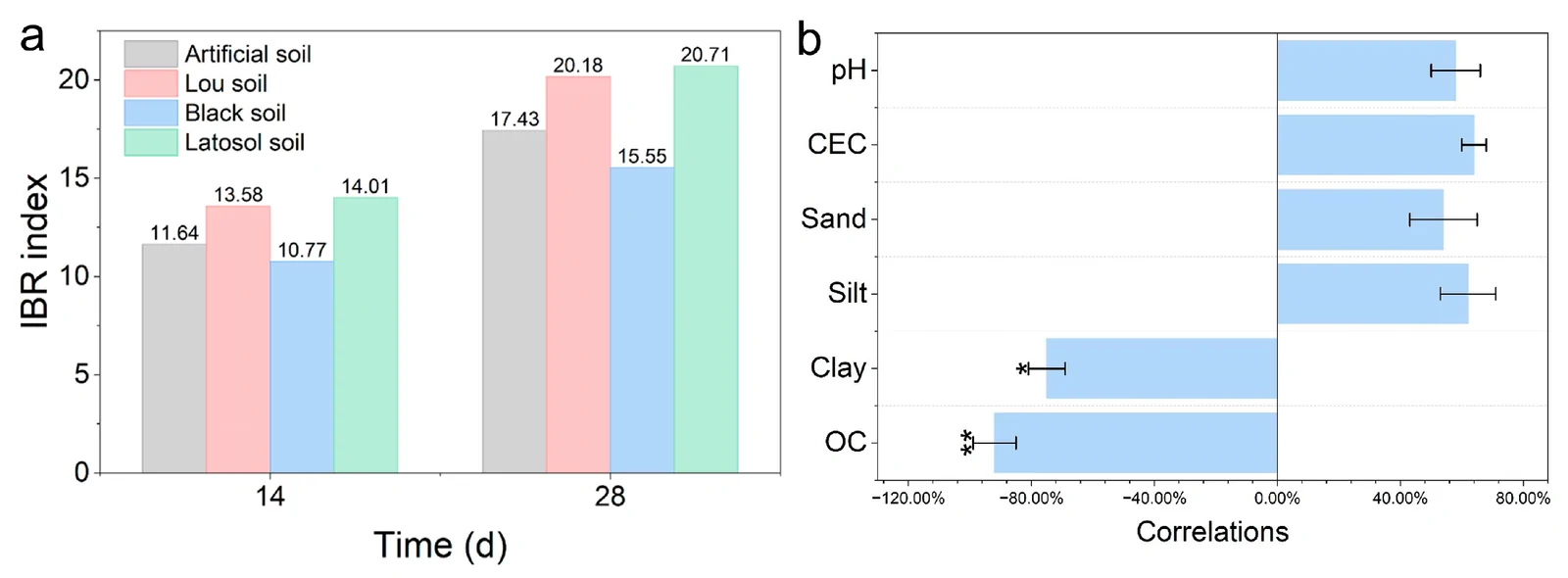
(**a**) The integrated biomarker response (IBR) index in four soils from 14 to 28 days of exposure, and (**b**) the correlation between soil properties and polystyrene (PS) toxicity. * and ** denote significant differences at *p* < 0.05 and *p* < 0.01, respectively.

The biological toxicity of PS in different soils was measured using the IBR index, which is commonly used to analyze the integrated toxicity effects at the cellular and tissue levels. In this study, responses to several biomarkers were assessed by IBR values based on exposure to 0.1 g kg^−1^ PS on day 14 and day 28 (Figure 8). At 0.1 g kg^−1^ PS, the measured biomarkers (ROS, SOD, CAT, GST, MDA, 8-OHdG) displayed significant physiological perturbations but without complete inhibition of antioxidant and detoxification enzymes. Higher concentrations (0.5, 1.0 g kg^−1^) led to irreversible enzyme inactivation, which would obscure the matrix-dependent differences in early stress responses that were the focus of this study. Using a low, sublethal dose maximizes the capacity to distinguish mild, soil-modulated toxic disparities among the four tested substrates. Sampling points at days 14 and 28 were chosen as day 14 marks the peak of antioxidant activation and day 28 reflects accumulated oxidative and genetic damage, together enabling comprehensive toxicity assessment via the IBR index. As shown in Figure 8a, the IBR values for artificial, lou, black, and latosol soils were 11.64, 13.58, 10.77, and 14.01 at day 14, respectively. Subchronic toxicity kept the same order at 28 d. However, IBR values increased by 44.4–49.7%, indicating greater toxicity over time. The IBR results further suggested the soil-dependent differences in the integrated toxic effects of PS on *Eisenia fetida*. Correlation analysis was further conducted to explore the relationship between soil physicochemical properties and PS toxicity (Figure 8b). The results showed that soil clay content and organic carbon (OC) were significantly negatively correlated with PS toxicity, indicating that higher OC and clay components were associated with the low bioavailability and low toxicity of PS.

## 4. Discussion

### 4.1. Soil Matrix Modulates the Acute Toxicity of PS

The 28 d LC_50_ values obtained from our toxicity assays suggested that PS lethal toxicity differs markedly across the four tested soil substrates. The lowest 28 d LC_50_ values (highest acute toxicity) occurred in lou and latosol soils, whereas black soil yielded the highest LC_50_ and weakest lethal impacts on *Eisenia fetida*. These experimental measurements validate our core experimental observation that soil matrix properties alter PS lethal hazard, consistent with prior ecotoxicological studies reporting that organic carbon and clay fractions modify contaminant bioavailability [24,27,28]. We hypothesize that black soil’s abundant organic carbon and well-developed soil aggregates may enhance PS adsorption and sequestration, limiting particle mobility and earthworm ingestion. This proposed matrix retention pathway aligns with published mechanistic work, but we emphasize that direct adsorption or aggregate binding measurements of PS were not conducted in the current study, so this interpretation remains a literature-supported hypothesis rather than empirically proven causation. By contrast, lou and latosol soils with low organic carbon content exhibit weaker capacity to retain PS particles, consistent with our measured pore water and tissue uptake datasets. It is critical to clarify that this causal chain linking low OC to elevated bioavailability is inferred from correlative bioavailability data, not confirmed via direct adsorption quantification. Soil matrix properties may also indirectly alter earthworm physiological stress responses, as documented in prior soil toxicity research [29]. Overall, exclusive reliance on toxicity data from artificial soil substrates cannot accurately reflect real-world terrestrial ecological hazards. Therefore, it is necessary to incorporate diverse representative natural soils as testing media in evaluating MP ecological risk. Notably, the concentrations used in the acute toxicity assays (ranging from 0 to 200 g kg^−1^) were selected to determine the LC_50_ values and establish the intrinsic toxicity thresholds of the PS particles. While these levels are higher than the concentrations typically reported in agricultural soils [25], such high-dose experiments are standard practice for deriving toxicological reference values required for ecological risk assessment. These results are hazard identification under worst-case scenarios rather than a simulation of current average environmental exposure. It is important to note that while the PS concentrations used in this study exceed typical levels found in agricultural soils, they provide a baseline for risk assessment models. Future research should focus on the long-term chronic effects of PS at environmentally relevant concentrations to fully elucidate the ecological risks.

### 4.2. Soil-Dependent Subchronic Toxicity of PS

PS exposure triggered excessive ROS accumulation in earthworms across all soil types, initiating a cascade of oxidative stress responses. This ROS overproduction subsequently disrupted the activities of the antioxidant defense system (SOD and CAT) and the detoxification enzyme GST. SOD activity exhibited a dual pattern of activation and inhibition, depending on both PS concentration and exposure duration. CAT activity followed a similar trend, likely because the hydrogen peroxide produced by SOD can inhibit CAT when it accumulates in large quantities [24]. The differential GST responses among soils further indicated the potential regulatory role of soil properties in PS toxicity. Ultimately, the sustained imbalance between ROS generation and the antioxidant capacity led to persistent lipid peroxidation, as indicated by the continuous increase in MDA content. This suggests that the earthworms’ self-regulation mechanisms were overwhelmed within the 28-day period. These results are consistent with previous studies on MP ecotoxicity [30,31]. The divergent antioxidant profiles measured across soil types correlate significantly with our experimentally determined pore water PS bioavailability and earthworm tissue PS accumulation values, establishing an empirical linkage between particle mobility and organismal oxidative stress intensity [32,33,34]. The observed discrepancies in PS bioavailability and earthworm uptake among the four soil types were likely associated with the distinct physicochemical properties governing PS behavior in the soil matrix [35]. As evidenced by the pore water analysis and earthworm uptake (Figure 7), black soil and artificial soil may mitigate the ecological risk of PS by reducing its bioavailability. The significantly larger PS particle sizes recovered from black soil and artificial soil suggest potential tendency for particle aggregation or flocculation [36,37]. This aggregation is likely driven by the high organic matter content and specific clay mineralogy characteristic of black soil, which enhances the adsorption and retention of PS particles within soil aggregates [27]. This aggregation/sequestration mechanism remains a speculative interpretation grounded only in particle size imaging and correlative bioavailability data; targeted adsorption isotherm, aggregate stability, or PS-soil binding experiments were not performed in this work to confirm causal binding relationships. Existing literature supports this hypothesized matrix interaction pathway [27,36,37], but we refrain from framing it as a definitive causal mechanism proven by our measurements. This stronger retention capacity likely restricts PS mobilization into pore water and reduces the particle pool accessible to earthworm ingestion [24], yet this interpretation remains merely a literature-supported hypothesis without direct empirical validation from our dataset. As a result, the reduced bioavailability may induce lower accumulation in earthworm tissues, potentially alleviating the toxic effects of PS in these environments [26]. Conversely, lou soil and latosol soil exhibit characteristics that exacerbate PS exposure and potential toxicity. The smaller PS particle sizes observed in these soils indicate weaker aggregation forces and higher stability of individual particles [27], leading to the significantly higher concentrations observed in pore water. For earthworms inhabiting lou and latosol soils, this increased mobility results in greater ingestion rates and subsequent bioaccumulation. Therefore, despite identical initial exposure concentrations, the environmental fate of PS in lou and latosol soils lead to a higher internal dose for earthworms, implying a heightened risk of adverse toxicological outcomes compared to black or artificial soils.

The content of 8-OHdG, a biomarker of DNA damage, was significantly higher in lou soil and latosol soil. Black soil with high organic matter content can adsorb PS particles and reduce their contact with earthworms. Meanwhile, soil organic matter can improve the antioxidant capacity of earthworms, scavenge excess ROS and alleviate DNA oxidative damage [24,36]. On the contrary, low organic matter content in lou soil and latosol soil weakened the immobilization effect on PS and the buffering capacity against oxidative stress, causing more serious genotoxicity.

Three-way ANOVA of subchronic biomarkers provided quantitative statistical evidence that soil type represented the primary determinant of PS toxic effects, outweighing the influences of PS concentration and exposure duration. The far larger F-statistics associated with soil type across ROS, antioxidant enzyme activity, lipid peroxidation and DNA damage indices suggested that inherent soil physicochemical characteristics override dose- and time-dependent toxic trends to shape the overall stress magnitude of *Eisenia fetida*. The IBR index, which integrates multiple biomarkers, further quantifies the overall toxic differences in PS in different soils. It is important to note that the IBR analysis in this study was conducted at a single exposure concentration. While this concentration was selected to represent environmentally relevant levels, it does not allow for the evaluation of dose-dependent responses. This limits the generalizability of the IBR values to other concentration levels, as the relative sensitivity of different biomarkers may vary with dose [26]. Additionally, the lack of concentration-dependent IBR trajectories precludes the assessment of whether the differences observed among soil types are consistent across lower exposure levels. Future studies incorporating a gradient of concentrations are recommended to fully characterize the concentration–response relationship of PS toxicity. The consistent toxicity ranking (latosol soil > lou soil > artificial soil > black soil) at two key exposure time points strongly supports the above conclusions. Black soil, with its high organic matter content and strong buffering capacity, exhibited the lowest IBR values, indicating the weakest integrated toxicity, while latosol and lou soils showed the strongest toxic effects. In contrast, artificial soil, which is widely used in standard ecotoxicological tests due to its reproducibility, failed to capture the true toxicity of PS in natural environments, as it cannot mimic the complex soil–PS interactions that govern PS bioavailability and the earthworms’ physiological responses. These findings highlight a critical limitation of relying solely on artificial soil toxicity tests to evaluate the ecological risks of MPs. Artificial soil tests often provide a simplified, uniform environment that does not reflect the heterogeneity of natural soils, leading to inaccurate assessments of PS toxicity. Therefore, to accurately assess the real ecological risks of MPs, it is essential to incorporate natural soil types with diverse physicochemical properties into toxicity tests, rather than relying solely on artificial soil.

Combined with correlation analysis results, we observed that soil organic carbon and clay content are significantly and negatively associated with PS toxicity. Higher OC and clay content contribute to the formation of organic–mineral complexes [36], which may wrap PS particles and further limit their migration in soil and reduce the probability of ingestion by earthworms. The number of soils investigated (*n* = 4) was limited for a substantive correlation analysis. The significant negative correlations between OC/clay and PS toxicity only reflect concurrent variation, rather than proving that OC or clay alone acts as an independent causal driver suppressing PS bioavailability and toxicity. It is likely that these properties, often covarying in natural soils, collectively influence the fate and toxicity of PS. For example, other physicochemical properties such as soil pH and cation exchange capacity (CEC) likely contribute to the observed differences in PS toxicity. As shown in Table S1, the four soils exhibited distinct pH and CEC values. Soil pH can influence the surface charge of both soil particles and PS, thereby affecting their electrostatic interactions, aggregation, and stability in the soil matrix [6,9]. Generally, PS particles tend to be negatively charged in environmental conditions. Thus, soil pH variations can alter the repulsive or attractive forces between PS and soil colloids. Furthermore, CEC, which reflects the soil’s capacity to hold exchangeable cations, is closely related to the content of clay minerals and organic matter. Soils with higher CEC generally possess a greater capacity to adsorb and retain particles and dissolved organic matter, potentially reducing the mobility and bioavailability of PS. The specific role in mediating the interactions between soil colloids and PS warrants attention. Therefore, the combined effects of soil properties likely create a complex matrix effect that governs the fate and toxicity of PS in terrestrial environments. Future studies incorporating a wider range of soils with divergent properties will be necessary to disentangle the specific contribution of each factor.

### 4.3. Implications and Limitations

The findings of this study have important practical implications for the ecological risk assessment of soil MPs. Currently, standard ecotoxicity tests for soil pollutants are mostly conducted using artificial soil, which has good reproducibility but cannot represent the heterogeneity of natural soil environments. Our results clearly show that relying solely on artificial soil tests may not reflect the actual toxicity of MPs in different natural soils, leading to biased risk assessment results. Therefore, it is necessary to incorporate representative natural soils with different physicochemical properties into the ecotoxicity test system of MPs.

Nevertheless, several key experimental limitations warrant explicit discussion. The present study was conducted using only one polymer type (polystyrene), one nominal particle size, one test organism (*Eisenia fetida*), and four distinct soil types. Consequently, the observed patterns of soil-dependent toxicity cannot be extrapolated to other MP types (e.g., polyethylene, polypropylene, or biodegradable plastics), other particle sizes (particularly nano-scale particles with different transport behaviors), other soil organisms with different exposure routes and physiological responses, or a broader range of soil physicochemical profiles. Systematic investigations incorporating a wider matrix of polymers, sizes, species, and soil types through meta-analytic approaches will be required before any general recommendations can be made regarding the suitability of artificial soil as a surrogate in MP risk assessment. Moreover, experimental assessment was confined to short-term acute and subchronic exposure regimes, whereas long-term multigenerational effects and community-level impacts were not evaluated. Future studies should extend exposure duration across multiple generations alongside soil biota community surveys to help achieve a more comprehensive evaluation of the long-term ecological risks posed by MPs. Meanwhile, direct measurements of PS adsorption, mobility, and aggregation behaviors in the soil matrix were not performed. The proposed mechanisms regarding soil–MP interactions are inferred from the observed bioavailability patterns and soil physicochemical characteristics. Future studies employing advanced imaging techniques or specific adsorption assays are needed to further verify these mechanistic hypotheses. More importantly, this study primarily focused on biochemical responses and acute lethality to elucidate the mechanisms of PS toxicity. While these biomarkers serve as sensitive early-warning signals of physiological stress, they do not fully capture the long-term ecological consequences at the population level. Endpoints such as growth, biomass variation, reproduction, and behavioral responses are critical for a holistic risk assessment. Future research should prioritize long-term chronic exposure experiments to investigate how soil matrix modulation affects the reproduction and growth of earthworms, thereby providing a more comprehensive evaluation of the ecological risks posed by MPs.

## 5. Conclusions

This study reveals a critical soil-dependent disparity in PS toxicity to earthworms. In comparison to artificial soil, the acute toxicity of PS appeared to be mitigated in black soil but exacerbated in lou and latosol soils. In addition, PS exposure was associated with elevated ROS levels in *Eisenia fetida*, which correlated with oxidative and DNA damage. Furthermore, the inhibitory effects of PS on antioxidant and detoxification enzymes were found to be soil-type-dependent. These results suggested that, under the experimental conditions of this study, toxicity estimates derived from artificial soil may not accurately reflect those in natural environments. Organic carbon and clay displayed significant negative correlations with PS toxicity within the four tested soils, suggesting a potential correlative linkage between high OC/clay and reduced PS bioavailability, though independent causal effects cannot be confirmed due to limited soil diversity and covarying soil properties. While follow-up validation using a broader range of MP polymers and test organisms is required, our findings highlight the potential bias introduced by standard testing substrates. Elucidating the specific roles of organic matter, clay minerals, and metal oxides in modulating PS toxicity across diverse soil environments represents a critical direction for future research.

## Figures and Tables

**Table 1:** The mortality and acute toxicity of PS in four soils.

PS Concentration (g·kg^−1^)	Initial Earthworm Number	Deaths Day 28			
		Artificial Soil	Lou Soil	Black Soil	Latosol Soil
0	30	0 ± 0	0 ± 0	0 ± 0	0 ± 0
10	30	2 ± 0	5 ± 0	1 ± 0	6 ± 1
25	30	4 ± 1	8 ± 1	3 ± 1	8 ± 0
50	30	9 ± 1	17 ± 1	8 ± 0	18 ± 1
100	30	13 ± 2	24 ± 2	10 ± 1	23 ± 1
200	30	18 ± 1	28 ± 1	16 ± 0	27 ± 2
28 d-LC_50_ (g·kg^−1^)		130.86 (88.15−254.27) ^a^	39.71 (29.66−52.12) ^a^	175.13 (113.74−398.06) ^a^	39.23 (28.24−52.99) ^a^

^a^ The values in brackets refer to 95% confidence intervals (CIs) of the mean.

## Data Availability

The original contributions presented in this study are included in the article/Supplementary Material. Further inquiries can be directed to the corresponding author.

## Supplementary Materials

The following supporting information can be downloaded at https://www.mdpi.com/article/10.3390/cimb48080780/s1.

## References

[1] de Souza Machado A.A., Kloas W., Zarfl C., Hempel S., Rillig M.C. (2018). Microplastics as an emerging threat to terrestrial ecosystems. Glob. Change Biol..

[2] Lau W.W.Y., Shiran Y., Bailey R.M., Cook E., Stuchtey M.R., Koskella J., Velis C.A., Godfrey L., Boucher J., Murphy M.B. (2020). Evaluating scenarios toward zero plastic pollution. Science.

[3] Wang Y., Bai R.H., Liu Q., Tang Q.X., Xie C.H., Richel A., Len C., Cui J.X., Yan C.R., He W.Q. (2025). Degradation of biodegradable plastic films in soil: Microplastics formation and soil microbial community dynamics. J. Hazard. Mater..

[4] Thompson R.C., Olsen Y., Mitchell R.P., Davis A., Rowland S.J., John A.W.G., McGonigle D., Russell A.E. (2004). Lost at sea: Where is all the plastic?. Science.

[5] Akanyange S.N., Lyu X., Zhao X., Li X., Zhang Y., Crittenden J.C., Anning C., Chen T., Jiang T., Zhao H. (2021). Does microplastic really represent a threat? A review of the atmospheric contamination sources and potential impacts. Sci. Total Environ..

[6] Mohasin M., Habib K., Rao P.S., Ahmad M., Siddiqui S. (2025). Microplastics in agricultural soils: Sources, impacts, and mitigation strategies. Environ. Monit. Assess..

[7] Tournier V., Topham C.M., Gilles A., David B., Folgoas C., Moya Leclair E., Kamionka E., Desrousseaux M.L., Texier H., Gavalda S. (2020). An engineered PET depolymerase to break down and recycle plastic bottles. Nature.

[8] Ji Q., Zhang Y., Xia Y., Wang X., He M., Yang Y., Sabel C.E., Huang B., Zhu F., Shao M. (2024). Centennial records of microplastics in Lake Cores in Huguangyan Maar Lake, China. Environ. Sci. Technol..

[9] Xu B.L., Liu F., Cryder Z., Huang D., Lu Z.J., He Y., Wang H.Z., Lu Z.M., Brookes P.C., Tang C.X. (2020). Microplastics in the soil environment: Occurrence, risks, interactions and fate—A review. Crit. Rev. Environ. Sci. Technol..

[10] Schefer R.B., Koestel J., Mitrano D.M. (2025). Minimal vertical transport of microplastics in soil over two years with little impact of plastics on soil macropore networks. Commun. Earth Environ..

[11] Egbeocha C.O., Malek S., Emenike C.U., Milow P. (2018). Feasting on microplastics: Ingestion by and effects on marine organisms. Aquat. Biol..

[12] Li Y.F., Ling W., Hou C., Yang J., Xing Y., Lu Q.B., Wu T.Q., Gao Z.Y. (2025). Global distribution characteristics and ecological risk assessment of microplastics in aquatic organisms based on meta-analysis. J. Hazard. Mater..

[13] Liu P., Lu K., Li J., Wu X., Qian L., Wang M., Gao S. (2020). Effect of aging on adsorption behavior of polystyrene microplastics for pharmaceuticals: Adsorption mechanism and role of aging intermediates. J. Hazard. Mater..

[14] Chen J.B., Huo L., Yuan Y., Jiang Y., Wang H., Hui K.L., Li Y.J., Huang Z.K., Xi B.D. (2024). Interactions between microplastics and heavy metals in leachate: Implications for landfill stabilization process. J. Hazard. Mater..

[15] Ma J.C., Cheng C., Du Z.K., Li B., Wang J.H., Wang J., Wang Z.B., Zhu L.S. (2019). Toxicological effects of pyraclostrobin on the antioxidant defense system and DNA damage in earthworms (*Eisenia fetida*). Ecol. Indic..

[16] Blouin M., Honson M.E., Delgado E.A., Baker G., Brussaard L., Butt K.R., Dai J., Dendooven L., Peres G., Tondoh J.E. (2013). A review of earthworm impact on soil function and ecosystem services. Eur. J. Soil Sci..

[17] Navarro I., Torre A.D.L., Sanz P., Porcel M.Á.P., Pro J., Carbonell G., Martínez M.D.L.Á. (2017). Uptake of perfluoroalkyl substances and halogenated flame retardants by crop plants grown in biosolids-amended soils. Environ. Res..

[18] Blakemore R.J., Hochkirch A. (2017). Restore earthworms to rebuild topsoil. Nature.

[19] Zhu L., Li B., Wu R.L., Li W.X., Wang J., Wang J.H., Du Z.K., Juhasz A., Zhu L.S. (2020). Acute toxicity, oxidative stress and DNA damage of chlorpyrifos to earthworms (*Eisenia fetida*): The difference between artificial and natural soils. Chemosphere.

[20] Yu Y.J., Li X.F., Yang G.L., Wang Y.H., Wang X.Q., Cai L.M., Liu X.J. (2019). Joint toxic effects of cadmium and four pesticides on the earthworm (*Eisenia fetida*). Chemosphere.

[21] Lichtfouse E., Chenu C., Baudin F. (1996). Resistant ultralaminae in soils. Org. Geochem..

[22] Song X.K., Lyu M.X., Zhang X.D., Ruthensteiner B., Ahn I.Y., Pastorino G., Wang Y., Gu Y., Ta K., Sun J. (2021). Large plastic debris dumps: New biodiversity hot spots emerging on the deep-Sea floor. Environ. Sci. Technol. Lett..

[23] Zhu K., Jia H., Zhao S., Xia T., Guo X., Wang T., Zhu L. (2019). Formation of environmentally persistent free radicals on microplastics under light irradiation. Environ. Sci. Technol..

[24] Liu J., Qin J., Zhu L., Zhu K., Liu Z., Jia H., Lichtfouse E. (2022). The protective layer formed by soil particles on plastics decreases the toxicity of polystyrene microplastics to earthworms (*Eisenia fetida*). Environ. Int..

[25] Lwanga E.H., Gertsen H., Gooren H.P.A., Peters P., Salanki T., Der Ploeg M.V., Besseling E., Koelmans A.A., Geissen V. (2016). Microplastics in the terrestrial ecosystem: Implications for *Lumbricus terrestris* (Oligochaeta, Lumbricidae). Environ. Sci. Technol..

[26] Sanchez W., Burgeot T., Porcher J.-M. (2013). A novel “integrated biomarker response” calculation based on reference deviation concept. Environ. Sci. Pollut. Res..

[27] Dissanayake P.D., Kim S., Sarkar B., Oleszczuk P., Sang M.K., Haque M.N., Ahn J.H., Bank M.S., Ok Y.S. (2022). Effects of microplastics on the terrestrial environment: A critical review. Environ. Res..

[28] Parus A., Lisiecka N., Klozinski A., Zembrzuska J. (2025). Do microplastics in soil influence the bioavailability of sulfamethoxazole to plants?. Plants.

[29] Gautam K., Dwivedi S., Verma R., Vamadevan B., Patnaik S., Anbumani S. (2024). Combined effects of polyethylene microplastics and carbendazim on *Eisenia fetida*: A comprehensive ecotoxicological study. Environ. Pollut..

[30] Wang X., Zheng H., Zhao J., Luo X.X., Wang Z.Y., Xing B.S. (2020). Photodegradation elevated the toxicity of polystyrene microplastics to grouper (*Epinephelus moara*) through disrupting hepatic lipid homeostasis. Environ. Sci. Technol..

[31] Li B., Song W.H., Cheng Y.L., Zhang K.H., Tian H.M., Du Z.K., Wang J.H., Wang J., Zhang W., Zhu L.S. (2021). Ecotoxicological effects of different size ranges of industrial-grade polyethylene and polypropylene microplastics on earthworms *Eisenia fetida*. Sci. Total Environ..

[32] Guo Z.Q., Li P., Yang X.M., Wang Z.H., Lu B.B., Chen W.J., Wu Y., Li G.W., Zhao Z.W., Liu G.B. (2022). Soil texture is an important factor determining how microplastics affect soil hydraulic characteristics. Environ. Int..

[33] Ren Z.F., Xu X.Y., Liang J., Yuan C.P., Zhao L., Qiu H., Cao X.D. (2024). Aging dynamics of polyvinyl chloride microplastics in three soils with different properties. Environ. Sci. Technol..

[34] Li J., Xu Z.H., Dai L.M., Cui M., Chen Y.J., Song Y., Wang S.S. (2025). Mineral-armored structure enhanced the stability of polyethylene microplastics rather than polylactic acid microplastics: A longterm natural aging study. Environ. Sci. Technol..

[35] Shao Y.T., Wang J.H., Wang J., Du Z.K., Li B., Zhu L.S., Juhasz A., Liu X.Y., Xu Y.Q., Li W.X. (2019). Oxidative stress and genotoxic effects in earthworms induced by five imidazolium bromide ionic liquids with different alkyl chains. Chemosphere.

[36] de Souza Machado A.A., Lau C.W., Till J., Kloas W., Lehmann A., Becker R., Rillig M.C. (2018). Impacts of microplastics on the soil biophysical environment. Environ. Sci. Technol..

[37] Qu M., Zhao X., Wang Q.A., Xu X., Chen H., Wang Y. (2024). PIEZO mediates a protective mechanism for nematode *Caenorhabditis elegans* in response to nanoplastics caused dopaminergic neurotoxicity at environmentally relevant concentrations. Ecotoxicol. Environ. Saf..

