# Supplementary material for: Soil Matrix Modulates Polystyrene Microplastic Toxicity to Eisenia fetida: From Acute Lethality to Oxidative Genotoxicity
Source: Curr Issues Mol Biol. 2026 Jul 30;48(8):780. doi: 10.3390/cimb48080780 (PMC13511216; doi:10.3390/cimb48080780)
Supplement: Supplementary file 1 [file cimb-48-00780-s001.zip › cimb-4410900-supplementary.pdf]

Soil matrix modulates polystyrene microplastic toxicity to *Eisenia fetida*: From acute lethality to oxidative genotoxicity

Xiaoling Xu[a], Chuanxiang Li[a], Haochen Sun[a], Jian Wang[a], Jinbo Liu[a]*,

[a] School of Petroleum Engineering and Environmental Engineering, Yan'an University, Yan'an, 716000, China

* Corresponding author E-mails: liujinbo@yau.edu.cn

Transparency Notice: The data presented in Tables S2–S5, and Figure S2 come from experiments newly performed after the original submission, rather than reserved unreported datasets from the initial trial. The original submission only contained data of 28 d acute lethality and subchronic biomarker detection.

Text S1 The contents of clay, silt and sand were detected using laser particle size analyzer (Mastersizer 2000E). Organic carbon (OC) was measured using the H_2SO_4-$K_2Cr_2O_7$ method. For metal contents, soil samples were completely digested by microwave using the Aqua Regia solution of 3/1 37%HCl/97%HNO_3. Concentration of metal ions in digestion solutions was measured by an inductively coupled plasma atomic emission spectrometer (ICP-AES) from Thermo-ICAP6300, Waltham. For pH determination, a 1/5 w/v mixture of soil in Milli-Q water with soil and solution was placed into a shaker for 1 h, then settled before measuring pH. Cation exchange capacity (CEC) was obtained by adding 0.2 M NH_4Cl to substitute exchangeable cations, such as K^+, Na^+, Ca^{2+}, Mg^{2+}, Al^{3+}, into supernatant from the three consecutive washes, then were analyzed by ICP-AES.

Text S2 The IBR index was calculated following the protocol of Sanchez et al. (2013). Specifically, Y_i is obtained by compare individual average biomarker data (X_i) with average reference data (X_0), in which logarithmic transformation is applied to decrease variance.

$$Y_i = \log (X_i / X_0)$$

By calculating the general mean (μ) and standard deviation (σ) of Y_i, a standardized biomarker response (Z_i) is obtained.

$$Z_i = (Y_i - \mu) / \sigma$$

In order to establish a basal line with 0 as the center and represent the changes of biomarkers based on this baseline, the Z_i and the average of the reference biomarker data (Z_0) are applied to define a biomarker deviation index (A_i).

$$A_i = Z_i - Z_0$$

The absolute value of A_i parameters for each biomarker are summed to obtain integrated biomarker response (IBR).

$$IBR = \sum |A_i|$$

Table S1. Physicochemical properties of four soils.

Soil type	pH	CEC (cmol kg^{-1})	OC (g kg^{-1})	Clay (%)	Silt (%)	Sand (%)
Artificial soil	6.00 ± 0.05	3.17 ± 0.48	25.13 ± 0.59	11.57	46.71	41.72
Lou soil	8.67 ± 0.07	37.73 ± 1.38	9.58 ± 0.68	12.79	55.42	31.79
Black soil	8.00 ± 0.20	31.32 ± 1.98	21.20 ± 0.68	13.10	45.01	41.89
Latosol soil	6.25 ± 0.05	13.92 ± 0.84	2.28 ± 0.39	9.82	22.56	67.62

OC*: organic carbon.

CEC**: cation exchange capacity.

Table S2. The mortality and acute toxicity of PS in four soils after 7 d exposure.

PS concentration ($g \cdot kg^{-1}$)	Initial earthworm number	Deaths day 7			
		Artificial soil	Lou soil	Black soil	Latosol soil
0	30	0 ± 0	0 ± 0	0 ± 0	0 ± 0
5	30	0 ± 0	0 ± 0	0 ± 0	0 ± 0
10	30	1 ± 0	2 ± 0	0 ± 0	3 ± 0
20	30	1 ± 0	3 ± 1	1 ± 0	3 ± 1
25	30	2 ± 1	4 ± 1	2 ± 0	4 ± 1
50	30	4 ± 1	8 ± 1	2 ± 0	9 ± 1
7 d-LC_{50} ($g \cdot kg^{-1}$)		310.81	119.48	412.69	115.58

Table S3. Three-way analysis of variance for biochemical responses in earthworms (Eisenia fetida) treated with polystyrene (PS).

Source of variation	df	ROS					SOD				
		SS	MS	F	P	η^2_p	SS	MS	F	P	η^2_p
Time	3	1562.41	520.8	52.34	<0.001	0.192	1316.28	438.76	48.72	<0.001	0.184
Dose	4	3485.67	871.42	87.62	<0.001	0.296	2958.65	739.66	82.19	<0.001	0.285
Soil	3	3789.25	1263.08	126.89	<0.001	0.378	3247.91	1082.64	118.42	<0.001	0.366
Time×Dose	12	2232.76	186.06	18.75	<0.001	0.14	1763.21	146.93	16.38	<0.001	0.126
Time×Soil	9	1985.78	220.64	22.64	<0.001	0.162	1634.53	181.61	20.17	<0.001	0.151
Dose×Soil	12	3577.22	298.1	29.81	<0.001	0.208	2606.18	217.18	27.05	<0.001	0.197
Time×Dose×Soil	36	3221.44	89.48	8.92	<0.05	0.096	2641.52	73.38	8.15	<0.05	0.089
Residual (Error)	640	6372.56	9.96	-	-	-	5748.37	8.98	-	-	-

Source of variation	df	CAT					GST				
		SS	MS	F	P	η^2_p	SS	MS	F	P	η^2_p
Time	3	1126.52	375.51	45.18	<0.001	0.176	968.34	322.78	42.65	<0.001	0.168
Dose	4	2653.78	663.45	79.43	<0.001	0.276	2315.12	578.78	76.08	<0.001	0.269
Soil	3	2815.64	938.55	112.65	<0.001	0.357	2467.89	822.63	108.37	<0.001	0.349
Time×Dose	12	1526.83	127.24	15.22	<0.001	0.118	1243.65	103.64	14.09	<0.001	0.111
Time×Soil	9	1423.77	158.2	18.93	<0.001	0.144	1164.86	129.43	17.25	<0.001	0.135
Dose×Soil	12	2392.45	199.37	25.37	<0.001	0.188	1897.26	158.11	23.16	<0.001	0.178
Time×Dose×Soil	36	2308.62	64.13	7.63	<0.05	0.084	1927.55	53.54	7.02	<0.05	0.078
Residual (Error)	640	5326.15	8.32	-	-	-	4865.29	7.6	-	-	-

Source of variation	df	MDA					8-OHdG				
		SS	MS	F	P	η^2_p	SS	MS	F	P	η^2_p
Time	3	746.28	248.76	38.91	<0.001	0.155	621.5	207.17	36.27	<0.001	0.148
Dose	4	1867.35	466.84	69.54	<0.001	0.251	1543.6	385.9	64.32	<0.001	0.239
Soil	3	1848.62	616.21	96.23	<0.001	0.322	1613.5	537.83	89.64	<0.001	0.31
Time×Dose	12	927.54	77.3	12.87	<0.001	0.103	831.2	69.27	11.56	<0.001	0.094
Time×Soil	9	936.81	104.09	15.62	<0.001	0.122	862.6	95.84	14.38	<0.001	0.113
Dose×Soil	12	1513.47	126.12	21.04	<0.001	0.165	1387.5	115.63	19.27	<0.001	0.155
Time×Dose×Soil	36	1689.25	46.92	6.58	<0.05	0.073	1529.2	42.48	6.14	<0.05	0.068
Residual (Error)	640	4258.73	6.65	-	-	-	3784.3	5.91	-	-	-

Notes: df = degrees of freedom; SS = Sum of Squares; MS = Mean Square; F = F-statistic; P = significance value; η^2_p = partial eta-squared (effect size). ROS, SOD, CAT, GST, MDA, and 8-OHdG represents reactive oxygen species, superoxide dismutase, catalase, glutathione S-transferase, malondialdehyde (MDA) and 8-hydroxydeoxyguanosine, respectively.

Table S4. The numerical dataset of PS in pore-water (items/L)

PS concentration ($g\ kg^{-1}$)	Artificial soil	Lou soil	Black soil	Latosol soil
0.1	1.2 ± 0.2 b	2.1 ± 0.3 a	1.1 ± 0.2 b	2.5 ± 0.3 a
0.25	2.8 ± 0.4 b	7.2 ± 0.8 a	1.9 ± 0.3 b	8.1 ± 0.9 a
0.5	7.1 ± 0.7 c	19.2 ± 1.1 a	7.3 ± 0.6 c	17.4 ± 1.0 b
1	18.0 ± 1.2 c	30.1 ± 1.3 b	14.8 ± 0.9 d	34.2 ± 1.5 a

n = 3 independent biological replicates per treatment; Values = Mean ± Standard Error; Different lowercase letters denote significant differences among soil types at the same PS concentration ($p < 0.05$).

Table S5. The numerical dataset of PS accumulation in earthworm tissues (items/g)

PS concentration ($g\ kg^{-1}$)	Artificial soil	Lou soil	Black soil	Latosol soil
0.1	3.0 ± 0.4 b	9.1 ± 0.8 a	3.2 ± 0.3 b	8.3 ± 0.7 a
0.25	5.2 ± 0.6 b	14.0 ± 1.2 a	6.0 ± 0.5 b	16.2 ± 1.3 a
0.5	8.9 ± 0.8 b	23.1 ± 1.5 ab	8.2 ± 0.6 b	27.0 ± 1.6 a
1	24.8 ± 1.8 b	41.2 ± 2.1 a	19.0 ± 1.2 c	43.5 ± 2.3 a

n = 3 independent biological replicates per treatment; Values = Mean ± Standard Error; Different lowercase letters denote significant differences among soil types at the same PS concentration ($p < 0.05$).

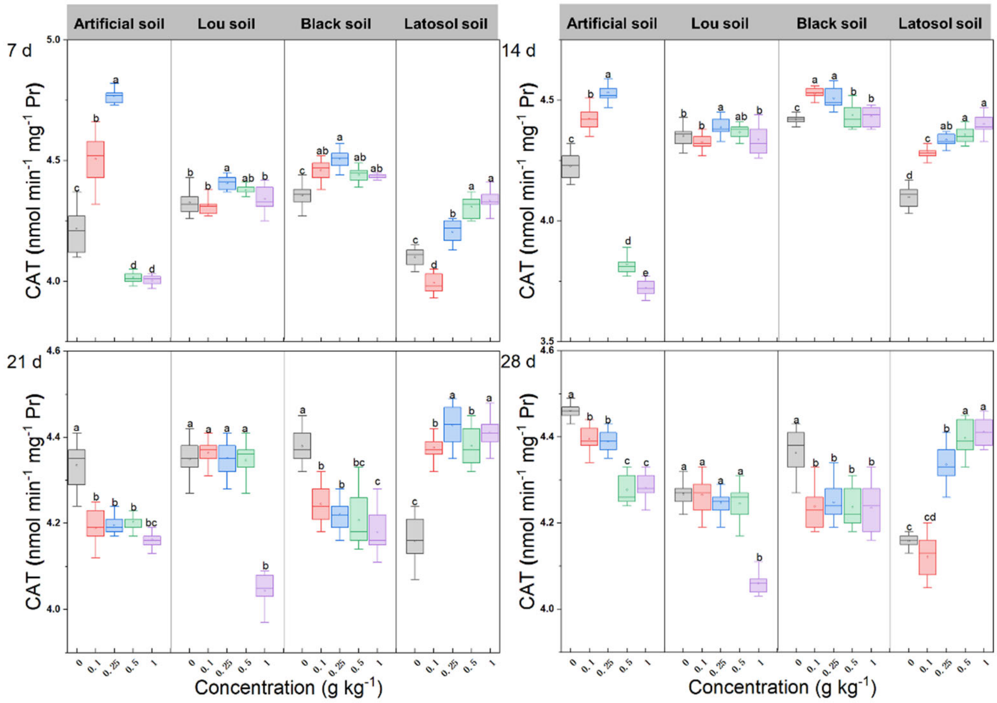

Figure S1. The catalase (CAT) activity in earthworms following polystyrene (PS) exposure at various concentrations in different soils. Different signals mean obvious differences between control and exposure treatments at $p < 0.05$ level.

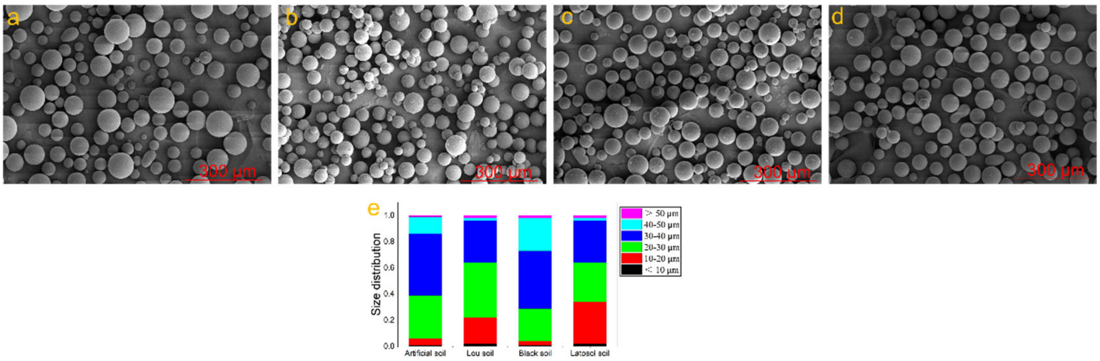

Figure S2. SEM images of PS exposed for 7 d in artificial soil (a), lou soil (b), black soil (c) and latosol soil (d), and the size distribution of PS in different soils (e). All particles in the SEM images were measured by using a Nano Measurer to obtain the size distribution.

References:

Sanchez, W.; Burgeot, T.; Porcher, J. A novel "integrated biomarker response" calculation based on reference deviation concept. *Environ. Sci. Pollut. Res.* **2016**, *20*, 2721–2725.
